# TRPA1 Activation-Induced Myelin Degradation Plays a Key Role in Motor Dysfunction After Intracerebral Hemorrhage

**DOI:** 10.3389/fnmol.2019.00098

**Published:** 2019-04-17

**Authors:** Min Xia, Weixiang Chen, Jie Wang, Yi Yin, Chao Guo, Chengcheng Li, Mingxi Li, Xiaoqin Tang, Zhengcai Jia, Rong Hu, Xin Liu, Hua Feng

**Affiliations:** Department of Neurosurgery, Southwest Hospital, Third Military Medical University, Chongqing, China

**Keywords:** transient receptor potential ankyrin 1 channel (TRPA1), myelin damage, nerve conduction, motor dysfunction, intracerebral hemorrhage

## Abstract

Intracerebral hemorrhage (ICH) is a devastating disease that is characterized by high morbidity and high mortality. ICH has an annual incidence of 10–30/100,000 people and accounts for approximately 10%–30% of all types of stroke. ICH mostly occurs at the basal ganglia, which is rich in nerve fibers; thus, hemiplegia is quite common in ICH patients with partial sensory disturbance and ectopic blindness. In the clinic, those symptoms are considered to originate from the white matter injury in the area, but the exact mechanisms are unknown, and currently, no effective drug treatments are available to improve the prognosis. Clarifying the mechanisms will contribute to the development of new treatment methods for patients. The transient receptor potential ankyrin 1 (TRPA1) channel is a non-selective cation channel that plays a role in inflammatory pain sensation and nociception and may be a potential regulator in emotion, cognition and social behavior. Here, we report that TRPA1 is involved in myelin damage and oxidative stress injury in a mouse ICH model. Intervention with the TRPA1 channel may be a new method to improve the motor function of patients in the early stage of ICH.

## Introduction

Intracerebral hemorrhage (ICH) comprises 10%–30% of all stroke cases (Balami and Buchan, 2012). After ICH, the case fatality rate is approximately 55% at 1 year and 70% at 5 years, and a few survivors regain functional independence (Joseph et al., 2016). Most instances of ICH occur in basal nuclei, which are rich in neural tracts (Zuo et al., 2017). Therefore, white matter injury is considered the major cause of hemiplegia with partial sensory disturbance and ectopic blindness, which are quite common symptoms in ICH patients. In fact, significant myelin and axonal injury were shown to occur in both an animal model and ICH patients. Severe myelin destruction and swelling axons are typical in the ICH animal model, but the exact mechanisms contributing to the loss of motor function are uncertain. This lack of understanding has hampered efforts to develop a therapeutic strategy aimed at protecting and repairing the damaged white matter of the ICH patients (Cheng et al., 2015; Tao et al., 2017).The transient receptor potential ankyrin 1 (TRPA1) channel is a type of nonselective transmembrane cation channel that contains a large number of N-terminal ankyrin repeats and has high permeability to calcium (Ca^2+^; De Logu et al., 2017). This channel is best known as a sensor of pain, cold, and environmental irritants and is expressed in sensory neurons, astrocytes, oligodendrocytes, ependymal cells located at ventricles and cardiomyocytes (Andrei et al., 2017; Lee et al., 2017). Recent studies have indicated that TRPA1 is involved in the myelin Ca^2+^ accumulation process and regulation of emotion, cognition, learning, memory, and social behavior (Hamilton et al., 2016; Lee et al., 2017). Previous research has shown that decreased pH and increased reactive oxygen species (ROS) production can activate the TRPA1, causing an inflow of Ca^2+^ (Andersson et al., 2008; Wang et al., 2011). The increase in Ca^2+^ activates NADPH oxidase 1 (NOX1), which acts by releasing oxidant molecules and increasing their expression, to produce more ROS, eventually exacerbating oxidative stress (Lee et al., 2009; De Logu et al., 2017). TRPA1 can promote the synaptic dysfunction mediated by the oligomeric forms of amyloid-β peptide in Alzheimer’s disease (AD; Bosson et al., 2017). TRPA1 deficiency has also been shown to attenuate cuprizone-induced demyelination (Sághy et al., 2016).

In this study, we investigated whether the TRPA1 channel was involved in white matter injury and motor function deficits in a mouse ICH model.

## Materials and Methods

### Animals

Adult male C57BL/6 mice (aged 8–10-weeks-old, 22–30 g) were used in this study. The mice were provided by the Experimental Animal Centre at the Third Military Medical University (Army Medical University). The mice were housed in a specific temperature-controlled room under a 12-h light/dark cycle and provided free access to food and water throughout the experimental period.

### ICH Model

All experimental protocols were approved by the Ethics Committee of the Third Military Medical University (Army Medical University) were performed according to the eighth edition of the National Institutes of Health Guide for the Care and Use of Laboratory Animals. The detailed procedures used to construct the ICH model were established in a previous study (Krafft et al., 2012). Briefly, the mice were anesthetized intraperitoneally with 1% phenobarbital sodium and were immobilized on the stereotaxic frame in a prone position. Then, a small cranial burr hole was made at a precise location in the mice (Bregma coordinates: 0.8 mm anterior and 2.1 mm lateral to the midline) under stereotactic guidance. The mice received 25 μl of autologous blood at a depth of 3 mm into the right basal ganglia at a rate of 2 μl/min using a sterile Hamilton syringe needle (Cat: RN1702) and a microinfusion pump (KD Scientific, Holliston, MA, USA). The mice were randomly divided into the following groups: Sham, ICH, ICH + HC-030031, ICH + A-967079, and ICH + polygodial. According to previous studies (Mendes et al., 2000; McNamara et al., 2007; Sałat and Filipek, 2015), the ICH mice received HC-030031 [30 mg/kg i.p. dissolved in isotonic saline with 4% dimethyl sulfoxide (DMSO) and 4% Tween 80], A-967079 (5 mg/kg s.c. dissolved in isotonic saline with 4% DMSO and 4% Tween 80) or polygodial (20 mg/kg s.c. dissolved in isotonic saline with 4% ethanol and 4% Tween 80) 1 h after the ICH procedure. On days 1, 3, 7, and 14 after surgery, the mice were euthanized, and their brains were collected for morphological and biochemical experiments. The TRPA1 antagonists HC-030031 and A-967079 and agonist polygodial were obtained from Sigma-Aldrich. According to the previous research method (Duncan et al., 2017), L-α-lysophosphatidylcholine purchased from Sigma-Aldrich was used to construct a demyelinating model under stereotactic guidance, which was used as a comparison of myelin morphology with that of the ICH mice. The mice received 2 μl of 1% L-α-lysophosphatidylcholine at a depth of 3 mm into the right basal ganglia at a rate of 50 nl/min using a sterile Hamilton syringe needle and a microinfusion pump. There were two preadministrated ICH groups to evaluate the effect of pretreatment (ICH* group represents the group given HC-030031 1 day before constructing ICH model, and ICH# group represents the group given A-967079 1 day before constructing ICH model).

### Immunofluorescence

The mice were deeply anesthetized with 1% pentobarbital sodium and transcardially perfused with 4% paraformaldehyde in 0.01 M phosphate-buffered saline (PBS) after an initial flush with isotonic saline. The brains were collected and post-fixed with 4% paraformaldehyde in 0.01 M PBS overnight, and then these tissues were dehydrated in 30% sucrose in 0.01 M PBS for 2 days. The brains were embedded in optimal cutting temperature compound (O.C.T. Compound, SAKURA, Whittier, CA, USA) and then sliced coronally (35 μm) on a cryostat microtome (CM1860UV, Leica, Wetzlar, Germany). For immunofluorescence staining, the cryostat floating sections were blocked with 5% bovine serum albumin (BSA) and 0.3% Triton X-100 for 1.5 h at room temperature and then sequentially incubated with primary antibodies overnight at 4°C and fluorescent secondary antibodies overnight at 4°C. The primary antibodies included rabbit anti-Neurofilament 200 (anti-NF200; 1:200; Sigma-Aldrich; Cat: N4142), goat anti-myelin basic protein (anti-MBP; 1:250; Santa Cruz; Cat: sc-13914), rabbit anti-degraded MBP (anti-DMBP; 1:250; Millipore; Cat: AB5864), rabbit anti-TRPA1 (1:250; Abcam; Cat: ab58844), rabbit anti-TRPA1 (1:250; Millipore; Cat: ABN1009), rabbit anti-NOX1 (1:500; Abcam; Cat: ab131088), and mouse anti-Calpain1 (1:250; Santa Cruz; Cat: sc-390677; Ottens et al., 2008; Deshmukh et al., 2013; Zou et al., 2017). The secondary antibodies included the following: AlexaFluor-488-, AlexaFluor-555-, or AlexaFluor-594-conjugated secondary antibodies against goat, rabbit and mouse (1:1,000; Invitrogen) antibodies. The nuclei were counterstained with 4′-6-diamidino-2-phenylindole (DAPI; Santa Cruz Biotechnology). Peri-hematoma region was defined as the surrounding white matter of the hematoma produced by the stereotactic injection of blood. Fluorescent images in the white matter adjacent to the hematoma were photographed using a Zeiss confocal microscope (Zeiss, LSM780).

### Western Blot Analysis

The western blot (WB) method was described in a previous study (Xie et al., 2017). The ipsilateral hematoma was removed by a magnifying glass using a microscopic instrument, and then the white matter surrounding the hematoma was collected for extraction. The entire process was completed at low temperatures. Equivalent amounts of a protein sample (50 μg) were loaded onto an SDS-PAGE gel after sample preparation. After gel electrophoresis was completed, the proteins were transferred to PVDF membranes, and the membranes were blocked in 5% BSA for 2 h at room temperature. Then, they were incubated with the following primary antibodies at 4°C overnight: goat anti-MBP (1:250; Santa Cruz; Cat: sc-13914), rabbit anti-NOX1 (1:500; Abcam; Cat: ab131088), mouse anti-Calpain1 (1:250; Santa Cruz; Cat: sc-390677), and rabbit anti-TRPA1 (1:2,000; Millipore; Cat: ABN1009; Das et al., 2013; Lee et al., 2017). β-actin was used as an internal loading control. Then, the membranes were probed with specific horseradish peroxidase-conjugated secondary antibodies (1:2,000; Invitrogen) for 2 h at room temperature. Finally, an enhanced chemiluminescence reagent kit (Thermo Scientific, Rockford, IL, USA) for western blotting was used to visualize the immunoreactive bands, which were detected with a bioimaging system (ChemiDoc XRS+; Bio-Rad, Hercules, CA, USA). The blot bands were quantified by densitometry using the Image Lab software (Image Lab 3.0; Bio-Rad, Hercules, CA, USA). Three animals per group and at least three repetitions of each treatment condition were used for the WB analysis.

### Transmission Electron Microscopy

For transmission electron microscopy (TEM), the animals were perfused with 1.25% glutaraldehyde and 2% paraformaldehyde in 0.1 M PB after an initial flush with isotonic saline. Then, the brains were rapidly removed and postfixed for at least 3 days at 4°C. The tissues were rinsed, postfixed with 1% OsO_4_ in PB for 2 h, counterstained with uranyl acetate, dehydrated with a graded acetone series, infiltrated with propylene oxide, and embedded in Epon. Ultrathin sections (~60 nm) were cut by using an ultramicrotome (LKB-V, LKB Produkter AB, Bromma) and observed under a transmission electron microscope (TECNAI10; Philips; Wang et al., 2018). A random image with 12 different fields of view was selected for each group for statistical analysis of the myelinated axons. The G-ratios of the myelinated fibers were calculated as the ratio of the axon diameter to the axon diameter with the myelin sheath using the ImageJ software (ImageJ 1.8; NIH, Bethesda, MD, USA), and at least 60 myelinated fibers per group were calculated (Chomiak and Hu, 2009). The grading system for focal myelin injury was described in a previous study (3 = complete myelin breakdown; 2 = myelin bubbling involving multiple lamella; 1 = one layer of myelin splitting; and 0 = normal compact myelin). A total of 100 myelinated fibers per group were used to assess myelin injury (Doyle et al., 2018). At least three mice per group were used in TEM analysis. The image of sham (D0) was derived from the tissue of the sham-operated group on the day of surgery, and the image of sham (D3) was derived from the tissue of the sham-operated group on the third day after surgery. The images of the two sham groups were used as controls for the treatment groups at the corresponding time points.

### Electrophysiologic Recording

To record the motor evoked potentials (MEPs) elicited by transcranial electrical stimulation, three mice per group were anesthetized with 1% pentobarbital sodium (25 mg/kg i.p.). Previous studies showed that this dose of pentobarbital sodium did not significantly affect the amplitude and waveform of MEPs in dogs and rodents (Redondo-Castro et al., 2016; Wu et al., 2017). The electrode placement was described in a previous study (Redondo-Castro et al., 2016). The stimulation needle electrodes were placed subcutaneously with the tip touching the scalp. The cathode was placed at the midpoint of an imaginary line connecting the two ears and the anode at the base of the nose. The hindlimbs of mice were exposed to enable insertion of recording electrodes into the gastrocnemius muscles. A ground electrode was placed subcutaneously in the back. Electrical stimulation was applied to excite the brain using a stimulator (Keypoint, Medtronic, USA). A single pulse of stimulation (7.8 mA, 0.1 ms, 1 Hz) was delivered *via* stimulation needle electrodes (DSN1620, Medtronic, USA). The electrical stimulation was repeated five times at intervals of 15 s in each mouse, and a trace of one of the five stimuli was presented as a representative in the figure (Supplementary Figure S1D). The trace represented the potential change that occurred after a single transcranial electrical stimulation, and two wave groups appeared after the stimuli. The two wave groups were regarded as the short-latency MEPs and the long-latency MEPs after transcranial electrical stimulation respectively. The short-latency MEPs were reported to involve brainstem reticulospinal tract, while long-latency MEPs mainly involved primary motor cortex and dorsal corticospinal tract (Wu et al., 2017). The long latency of MEPs was recorded for analysis, and it was measured from the onset of the transcranial electrical stimulation to the beginning of the evoked-event. An average long latency of these five stimuli was used for statistics. D-1 indicates the day before the ICH surgery, and D0 indicates the day the mice underwent ICH surgery.

### Behavioral Assessments

All tests were monitored by a digital video camera and analyzed in a blinded manner. The mice were weighed before the daily behavioral test. The behavioral tests were performed at the specified time, during the light cycle. D-1 indicates the day before the ICH surgery, and D0 indicates the day the mice underwent ICH surgery.

### Beam Balance Test

The mice had to cross a round wooden beam that was 1.5 cm in diameter and 70 cm in length and was elevated 30 cm from the ground for 1 min. The score (0–4) was decided by the walking distance. The average score of three consecutive trials was calculated (Schneider et al., 2016; Yin et al., 2017).

### Basso Mouse Scale (BMS)

This open-field locomotor scoring system consisted of scores from 0 (no ankle movement) to 9 (frequent or consistent plantar stepping, mostly coordinated, paws parallel at initial contact and lift off, normal trunk stability and tail always up; Wu et al., 2017).

### Mouse Forelimb Muscle Strength

The mouse forelimb muscle strength was detected by the Grip Strength Meter (1027SM Columbus Instruments).

### Statistical Analysis

All statistical analyses were performed using the SPSS 18.0 software. The data are expressed as the mean ± SEM. Comparisons between two groups were analyzed using a two-tailed Student’s *t*-tests. The behavioral data, weights and electrophysiological data from the different groups collected at different time points were analyzed using a mixed design two-way ANOVA. The G-ratio, WB results, fluorescence intensity and axon diameters were analyzed using one-way repeated measures ANOVA, followed by Scheffe’s *post hoc* test. The survival rate was analyzed by log-rank (Mantel-Cox) test. Differences were considered statistically significant at *P* < 0.05.

## Results

### Neuronal Tract Damage and Motor Dysfunction in the ICH Mice

**Figure 1:**
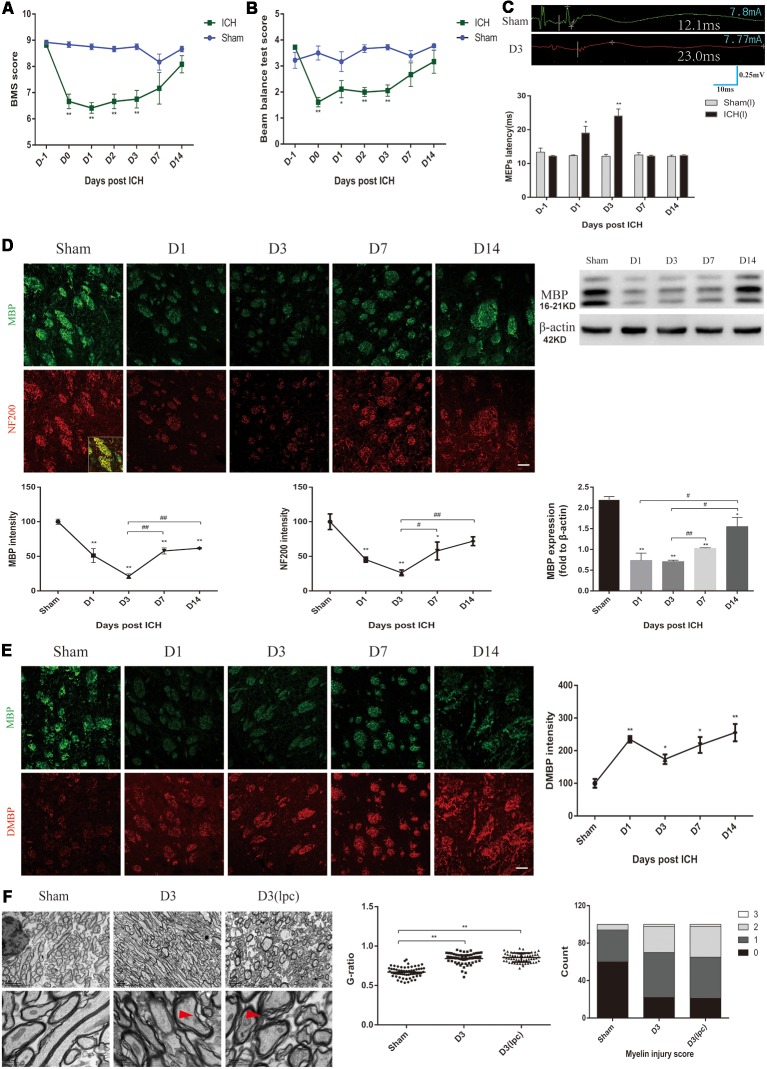
Motor dysfunction, nerve conduction damage and histological changes of the intracerebral hemorrhage (ICH) mice. **(A,B)** The behavioral scores of the ICH mice first decreased and then gradually increased after ICH (*n* = 6). **(C)** The motor evoked potentials (MEPs) latency was prolonged on days 1 and 3 after ICH, and the left MEPs was detected (*n* = 3). **(D)** Myelin basic protein (MBP) and neurofilament 200 (NF200) expression were significantly reduced in the acute phase of ICH (*n* ≥ 3). **(E)** Degraded MBP (DMBP) expression was increased after ICH (*n* ≥ 3). **(F)** After ICH, transmission electron microscopy (TEM) showed that the myelin sheath around the hematoma thinned and disintegrated. The time before white vertical lines represents the long latency of MEPs, and the moment of using electrical stimulation is at the beginning of the trace. Crossbars are the markers of the amplitude of MEPs detected by software. Unless specified, scale bars represent 50 μm. The red arrows mark the phenomenon of demyelination (*P < 0.05 vs. sham group, **P < 0.01 vs. sham group, ^#^*P* < 0.05, ^##^*P* < 0.01).

In this study, changes in locomotion, muscle strength and balance in the ICH mice were characterized by using various behavioral mouse models. First, compared with those of the sham group mice, the ICH mice showed a lower score in the basso mouse scale (BMS) and beam balance tests on D0-D3 (Figures 1A,B). The forelimb muscle strength of the ICH mice was lower than that of the sham group mice on D0-D3 (Supplementary Figure S1A). However, no significant differences were observed in the BMS score, the beam balance test score and forelimb muscle strength between the two groups on D7 and D14 (Figures 1A,B; Supplementary Figure S1A). No significant difference in weight was noted between the two groups throughout the experimental period (Supplementary Figure S1B). The MEPs latency was used to test nerve conduction function (Figure 1C). In contrast to that of the sham group mice, the latency of the ICH mice was significantly extended on D1 and D3 and returned to the sham group level on D7 and D14 (Figure 1C). These results suggested the ICH mice had significant motor dysfunction and nerve conduction damage during the acute phase of ICH.

MBP, DMBP and NF200 were used to assess the condition of the white matter after ICH. The coronal sections showed a progressive, overt decrease based on staining for normal MBP expression in the peri-hematoma region on D1 and D3 after ICH, and the immunofluorescence gradually recovered on D7 and D14 (Figure 1D). MBP protein expression showed a similar trend (Figure 1D). Simultaneously, the DMBP immunofluorescence was enhanced after ICH, which indicated that myelin degradation was increased (Figure 1E). Moreover, the decrease in NF200 was parallel with that of MBP (Figure 1D). Myelin damage can lead to slowed or reduced action potential conduction (Armstrong et al., 2016). Therefore, the severe myelin degradation of the ICH mice may be related to the prolonged MEPs latency.

Then, the white matter of the sham group mice and ICH group mice was compared using TEM. We noted oedema of the axons and disintegration of myelin in the peri-hematoma white matter, and an increased G-ratio indicated thinning of the myelin sheath (Figure 1F, Supplementary Figure S1C). The demyelinating model is a classic myelin injury model (Plemel et al., 2018). The myelin sheath of the ICH mice was compared to that of the demyelinating model, which was generated by stereotactically injecting L-α-lysophosphatidylcholine into the inner capsule. Interestingly, the pathological changes of the white matter were similar, probably because the two models shared some common myelin damage signaling pathways (Figure 1F). These results indicated severe neuronal tract damage and motor dysfunction in the acute phase after ICH in mice.

### Improved Nerve Conduction and Behavioral Performance of the ICH Mice After TRPA1 Blockade

**Figure 2:**
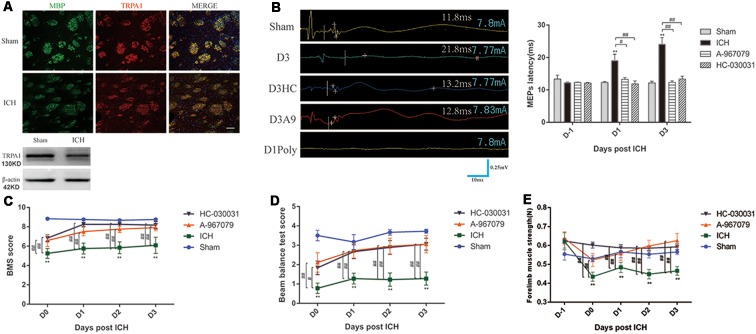
Transient receptor potential ankyrin 1 (TRPA1) blockers improved motor dysfunction and nerve conduction in the ICH mice. **(A)** TRPA1 was expressed on the myelin. The sham group was assessed on D1 after sham surgery. **(B)** The extended MEPs latency was restored by TRPA1 blockers, and the left MEPs was detected (*n* = 3). **(C,D)** The TRPA1 blockers (HC-030031 and A-967079) increased the basso mouse scale (BMS) and beam balance test scores of the ICH mice (*n* = 6). **(E)** The TRPA1 blockers improved muscle strength in the ICH mice (*n* = 6). Unless specified, scale bars represent 50 μm. The time before white vertical lines represents the long latency of MEPs, and the moment of using electrical stimulation is at the beginning of the trace. Crossbars are the markers of the amplitude of MEPs detected by software (**P < 0.01 vs. sham group, ^#^*P* < 0.05, ^##^*P* < 0.01).

TRPA1 expression on myelin was investigated. The immunofluorescence and WB analyses demonstrated that TRPA1 was expressed in the white matter and was co-labeled with the MBP (Figure 2A, Supplementary Figure S2A). The TRPA1 blockers (HC-030031 and A-967079) and the agonist polygodial were applied to the ICH mice to observe their impacts on the MEPs latency. The experimental results showed that HC-030031 and A-967079 shortened the MEPs latency of the ICH mice on D1 and D3. In contrast, in the ICH mice using the TRPA1 agonist polygodial, transcranial electrical stimulation did not induce MEPs (Figure 2B). Moreover, polygodial significantly increased the mortality of the ICH mice. The first three mice given TRPA1 agonist died in the afternoon after constructing ICH model (about 6 h after surgery), one mouse died on the second and third day respectively, and no mice died after 3 days. There was a significant difference in survival rate between the two groups (Supplementary Figure S2B). Further research indicated that HC-030031 and A-967079 obviously improved the BMS, beam balance test and the forelimb muscle strength scores in the first 3 days of ICH (Figures 2C–E). But there was no significant difference between the preadministrated ICH group and the ICH group, except for ICH group and ICH* group on D3 in the forelimb muscle strength test (Supplementary Figure S2C). The above findings suggested that TRPA1 blockers could significantly ameliorate nerve conduction and motor dysfunction during the acute phase in ICH mice.

### Improvement of the Neuronal Tract After Application of a TRPA1 Blocker in the ICH Mice

**Figure 3:**
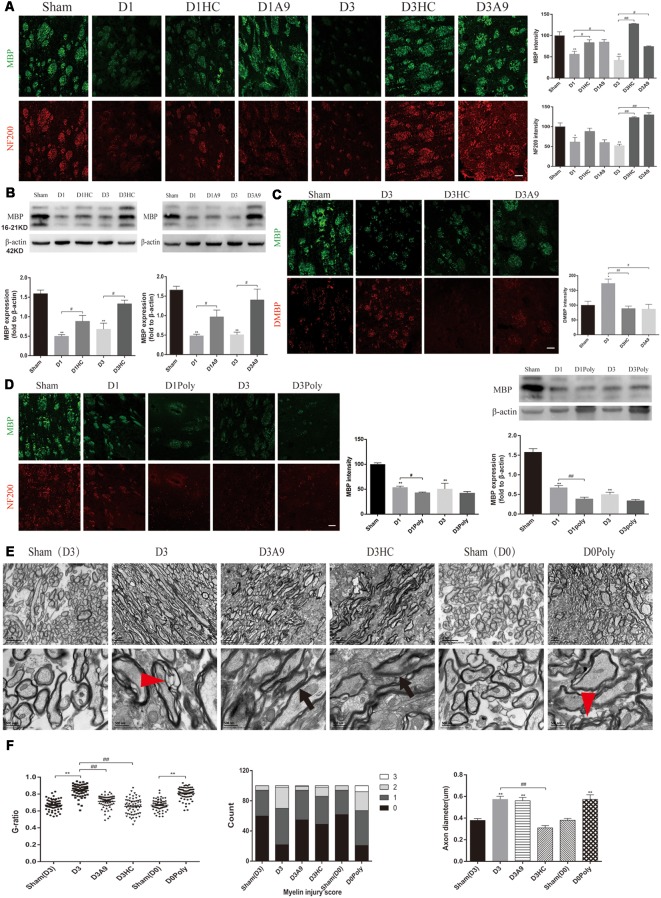
Changes in the white matter after application of the TRPA1 blockers and agonist to the ICH mice. **(A)** The TRPA1 blockers significantly enhanced the immunofluorescence intensity of NF200 and MBP on D1 and D3 in the ICH mice (*n* ≥ 3). **(B)** MBP expression was increased after application of the TRPA1 blockers. The sham group was assessed on D3 after sham surgery (*n* ≥ 3). **(C)** The TRPA1 blockers reduced the immunofluorescence intensity of DMBP on D3 in the ICH mice (*n* ≥ 3). **(D)** The TRPA1 agonist aggravated the myelin damage. The sham group was assessed on D3 after sham surgery (*n* ≥ 3). **(E)** The thinning and disintegration of myelin were reduced by the TRPA1 blockers. **(F)** The TRPA1 blocker HC-030031 could reduce axonal oedema. The red arrows mark the phenomenon of demyelination, and the black arrows indicate the reduction of demyelination. Unless specified, scale bars represent 50 μm (*P < 0.05 vs. sham group, **P < 0.01 vs. sham group, ^#^*P* < 0.05, ^##^*P* < 0.01).

The NF200 and MBP immunofluorescence were significantly enhanced on D1 and D3 after HC-030031 and A-967079 application in the ICH mice (Figure 3A). The improved MBP protein expression indicated that HC-030031 and A-967079 reduced the degradation of myelin skeletal proteins (Figure 3B). The reduced fluorescence intensity of DMBP showed that HC-030031 and A-967079 also reduced MBP degradation (Figure 3C). Conversely, we found that polygodial deteriorated myelin damage with a decreased fluorescence intensity and reduced MBP expression on D1 (Figure 3D). TEM showed that HC-030031 and A967079 reduced the degree of demyelination in the acute phase of ICH, leading to a decline in the G-ratio and the improvement of the myelin injury score (Figure 3E). Additionally, HC-030031 could ameliorate the axonal oedema (Figure 3F), whereas application of polygodial showed severe myelin damage and axonal oedema (Figures 3E,F). These findings suggested that TRPA1 played a vital role in myelin damage after ICH.

### TRPA1-Induced Myelin Degradation Was Mediated by Upregulation of NOX1 and Calpain1

**Figure 4:**
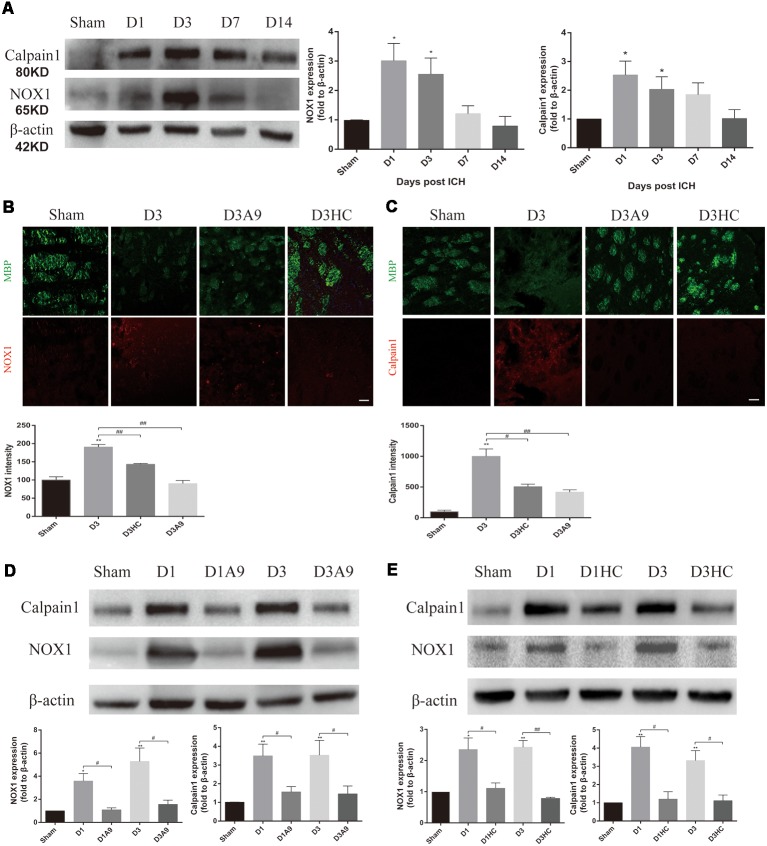
The signal pathway downstream of TRPA1 induced myelin injury. **(A)** NADPH oxidase 1 (NOX1) and Calpain1 expression were enhanced in the ICH mice. The sham group was assessed on D3 after sham surgery (*n* ≥ 3). **(B,C)** The TRPA1 blockers reduced the immunofluorescence intensity of NOX1 and Calpian1 on D3 in the ICH mice (*n* ≥ 3). **(D,E)** NOX1 and Calpain1 expression were reduced in the ICH mice after application of the TRPA1 blockers. The sham group was assessed on D3 after sham surgery (*n* ≥ 3). Scale bars represent 50 μm (*P < 0.05 vs. sham group, **P < 0.01 vs. sham group, ^#^*P* < 0.05, ^##^*P* < 0.01).

Considerable amounts of oxygen free radicals are released after ICH and may activate the TRPA1 signaling pathway (Qureshi et al., 2009; Qu et al., 2016). Therefore, we examined the NOX1 level after ICH and the changes in NOX1 expression after the application of TRPA1 blockers. The results demonstrated that NOX1 expression was increased after ICH (Figures 4A,B). A significant decrease of NOX1 was observed after application of HC-030031 and A-967079 (Figures 4B,D,E).

Moreover, according to previous studies, elevated Ca^2+^ can activate Calpain1 and increase its expression; this protein is involved in myelin degradation (Liu et al., 2006; Qureshi et al., 2009; Zhao et al., 2017; Baraban et al., 2018). Hence, changes in Calpain1 expression in the ICH mice were examined before and after use of the TRPA1 blockers. The results implied that Calpain1 expression was increased in the ICH mice (Figures 4A,C) and that its expression was significantly down-regulated after administration of HC-030031 and A-967079. These findings proved that TRPA1 blockers could reduce the amount of Calpain1 and thus reduce MBP degradation (Figures 4C–E). These results indicated that up-regulation of NOX1 and Calpain1 mediated the myelin degradation induced by TRPA1 activation. The TRPA1 blockers reduced the oxidative stress response of the white matter and the myelin damage after ICH.

## Discussion

Although white matter injury has long been considered the major cause of hemiplegia, partial sensory disturbance and ectopic blindness in ICH patients, our understanding of the mechanisms of white matter injury is still quite limited. Indeed, little is known about the effects on the neural fibers in the damaged area (Tao et al., 2017).

**Figure 5:**
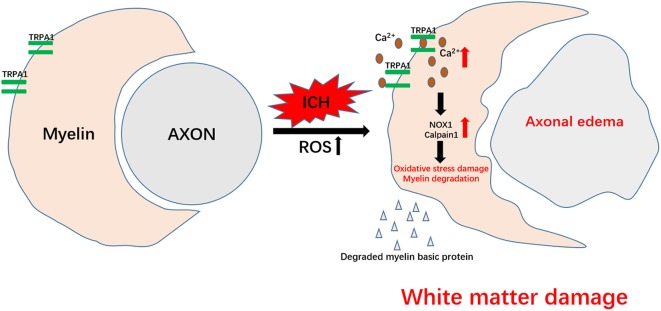
Schematic representation of TRPA1-mediated myelin damage after ICH. TRPA1 is activated by the increased reactive oxygen species (ROS) after ICH, leading to an increase in the TRPA1-mediated Ca^2+^ influx. The increased Ca^2+^ further contributes to the rise in NOX1 and Calpain1, causing oxidative stress damage and myelin degradation. Axons that lose myelin protection are more susceptible to ICH injury. The damaged white matter eventually aggravates motor dysfunction in the mice.

In this study, we observed that the neuronal tract damage and motor dysfunction of the ICH mice were severe in the acute phase and gradually improved after 7 days. The nonselective TRPA1 cation channel was involved in myelin injury after ICH, and inhibition of TRPA1 during the acute phase of ICH could ease the peri-hematoma white matter injury, nerve impulse conduction disorder and motor dysfunction in the mice. However, the preadministration of TRPA1 antagonism did not produce an obvious protective effect on motor function in ICH mice. This may be due to the metabolism of the TRPA1 antagonism by the mouse, resulting in the TRPA1 antagonism for pretreatment that is insufficiently concentrated after ICH to provide protection. ROS increases have been widely reported in ischemic stroke and hemorrhagic stroke, and the increase in ROS has also been found to activate TRPA1 (Hamilton et al., 2016; Qu et al., 2016; Arenas et al., 2017; Figure 5).

Axonal injury and demyelination at the edge of the hematoma were first described in a collagenase-induced ICH rat model (Wasserman and Schlichter, 2008). Significant demyelination and axonal damage were observed on D3 after ICH and were highly associated with motor dysfunction. Our group and others observed the same phenomena in the autologous blood infusion ICH mouse model (Barratt et al., 2014; Tao et al., 2016). Our data showed that the white matter injury severity peaked on D3 after ICH together with the obvious motor function deficits detected with several motor function tests; however, the axon demyelination lasted at least 2 weeks after ICH, whereas most of the motor function tests returned to normal on D7 after ICH. Data from other groups were consistent with our observation (Ni et al., 2015; Yang et al., 2018). Most of the motor function tests used here were adapted from an ischemic stroke mouse model; therefore, we assumed that the discordance of demyelination with the motor function tests was partially caused by inadequate sensitivity of the test. We developed a modified motor function test that could distinguish motor function deficits on day 28 after ICH [modified behavioral tests to detect white matter injury-induced motor deficits after intracerebral hemorrhage in mice (under review)]. Therefore, the improved function test could more accurately assess the relationship between white matter injury and motor dysfunction.

Using TEM to evaluate the white matter damage, we found that the morphological changes of the axons in the ICH mice were almost the same as those in the L-α-lysophosphatidylcholine-induced demyelinating mice. Sághy et al. (2016) and Bölcskei et al. (2018) showed that cuprizone-treated TRPA1 KO animals had reduced myelin damage and attenuated accumulation of astrocytes and microglia in 2016 and 2018, respectively. In the absence of the TRPA1 receptor, the release of astrocyte factors is altered, which significantly increases ERK1/2 and decreases both p38-MAPK and c-Jun activation, resulting in less oligodendrocyte apoptosis (Sághy et al., 2016; Bölcskei et al., 2018). Our results suggested that the increases in NOX1 and Calpain1 were associated with TRPA1 activation-induced myelin degradation and that blockade of TRPA1 could reduce NOX1 and Calpain1 expression. These studies also demonstrate that the myelin and oligodendrocyte damage begins with the increase in Ca^2+^ that is apparent with TRPA1. Together, these investigations indicate that TRPA1 is involved in myelin damage through a variety of mechanisms. In addition, destruction of the myelin sheath was more serious and the mortality rate of the ICH mice was distinctly increased by the TRPA1 agonist, the related mechanism needs further exploration. Collectively, interventions for TRPA1-mediated myelin damage may represent a new and hopeful target to save patients’ motor functions.

## Ethics Statement

All experimental protocols were approved by the Ethics Committee of the Third Military Medical University (Army Medical University) and performed according to the eighth edition of the National Institutes of Health Guide for the Care and Use of Laboratory Animals.

## Author Contributions

HF and XL were responsible for experimental design. MX and WC conducted the experiments and wrote the manuscript. RH, YY, JW, CG, XT, CL, and ML collected the data. XL and ZJ revised the manuscript. MX and WC have contributed equally to this work.

## Conflict of Interest Statement

The authors declare that the research was conducted in the absence of any commercial or financial relationships that could be construed as a potential conflict of interest.

## Supplementary Material

FIGURE S1 The changes in muscle strength, weight and the axons of the ICH mice. **(A)** The muscle strength was lower in the ICH mice than that in the sham group mice on D0–D3 (*n* = 6). **(B)** Comparison of weights between the two groups did not show a significant difference (*n* = 6). **(C)** Axonal oedema was observed around the hematoma in the ICH mice. **(D)** The five traces of D3 as an example of the MEPs approach (**P* < 0.05 vs. sham group, ***P* < 0.01 vs. sham group).

FIGURE S2 **(A)** TRPA1 was expressed on the myelin. The sham group was on D1 after sham surgery. **(B)** The TRPA1 agonist (polygodial) significantly increased the mortality of the ICH mice (*n* = 8; *P* < 0.05). **(C)** There was no significant difference between the preadministrated ICH group and the ICH group, except for ICH group and ICH* group on D3 in the forelimb muscle strength test (*n* = 3). Scale bars represent 50 μm (^#^*P* < 0.05).
