# Supplementary figures and images for: TRPA1 Activation-Induced Myelin Degradation Plays a Key Role in Motor Dysfunction After Intracerebral Hemorrhage
Source: Front Mol Neurosci. 2019 Apr 17;12:98. doi: 10.3389/fnmol.2019.00098 (PMC6478672; doi:10.3389/fnmol.2019.00098)

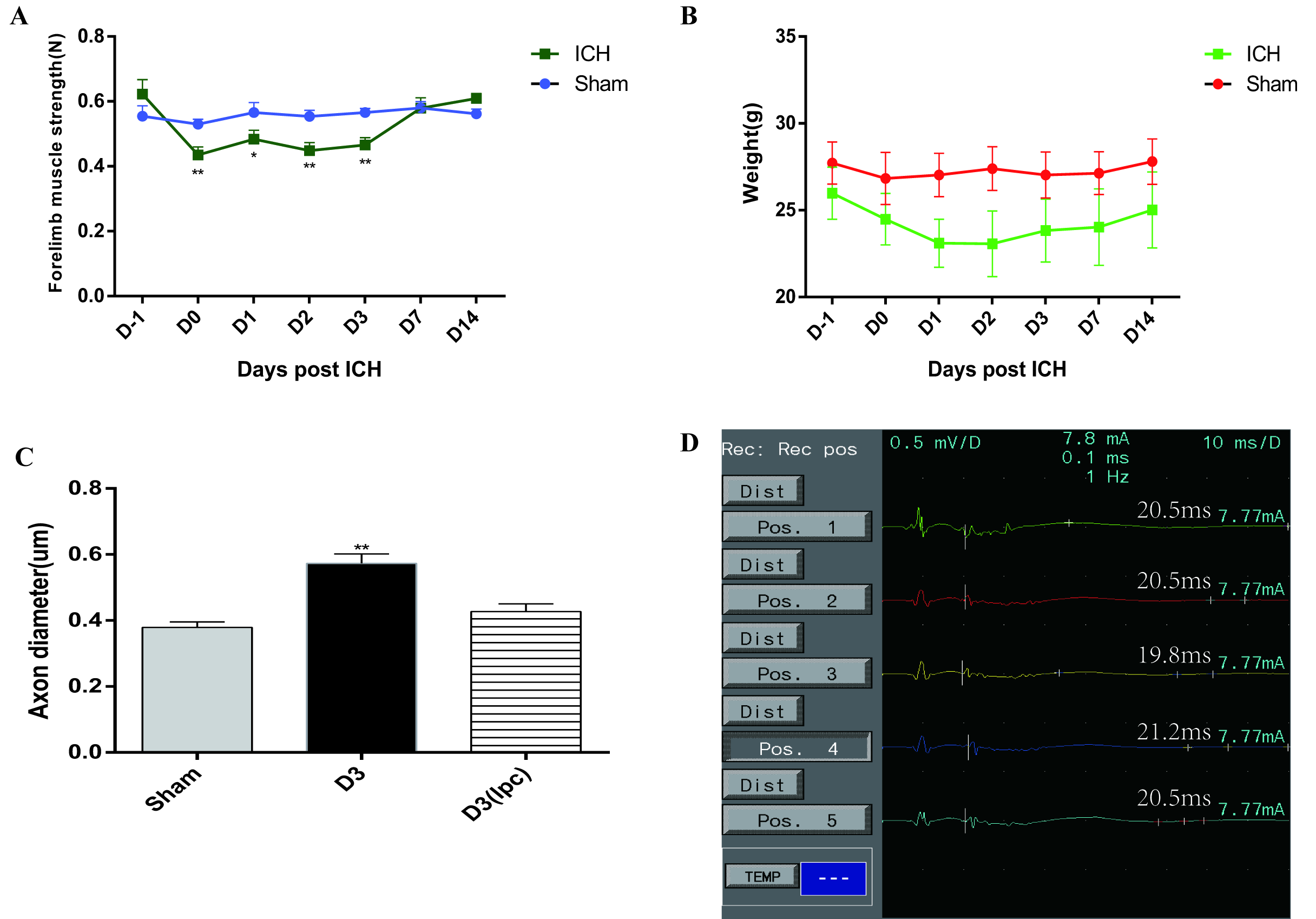

Supplement: FIGURE S1 — The changes in muscle strength, weight and the axons of the ICH mice. (A) The muscle strength was lower in the ICH mice than that in the sham group mice on D0–D3 (n = 6). (B) Comparison of weights between the two groups did not show a significant difference (n = 6). (C) Axonal oedema was observed around the hematoma in the ICH mice. (D) The five traces of D3 as an example of the MEPs approach (*P < 0.05 vs. sham group, **P < 0.01 vs. sham group). [file Image_1.TIF]

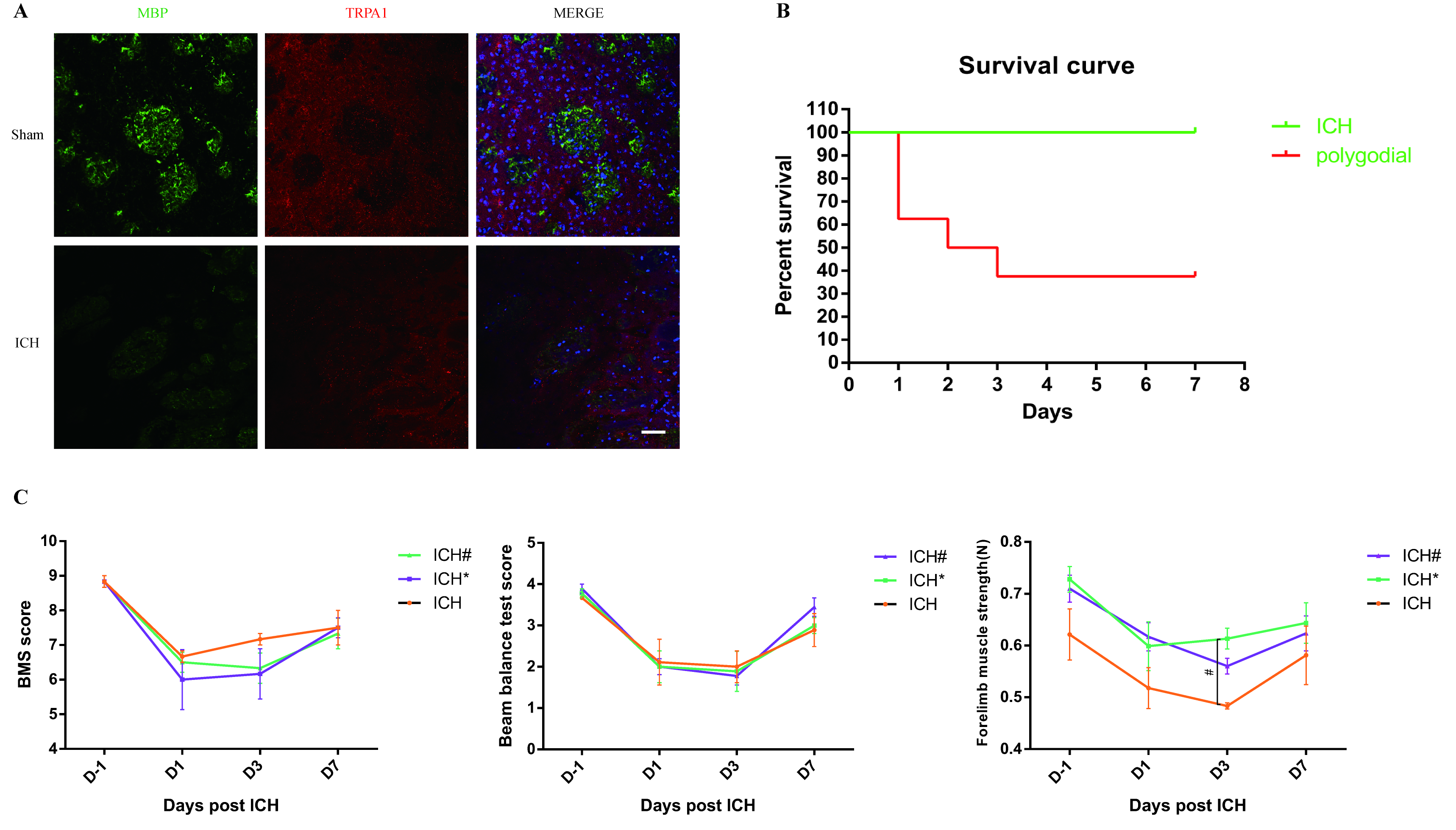

Supplement: FIGURE S2 — (A) TRPA1 was expressed on the myelin. The sham group was on D1 after sham surgery. (B) The TRPA1 agonist (polygodial) significantly increased the mortality of the ICH mice (n = 8; P < 0.05). (C) There was no significant difference between the preadministrated ICH group and the ICH group, except for ICH group and ICH* group on D3 in the forelimb muscle strength test (n = 3). Scale bars represent 50 μm (#P < 0.05). [file Image_2.TIF]
